# Thalidomide modulates macrophage-mediated inflammatory innate immune response during *Klebsiella pneumoniae *B5055 infection in BALB/c mice

**DOI:** 10.1186/cc11690

**Published:** 2012-11-14

**Authors:** V Kumar, S Chhibber

**Affiliations:** 1 Panjab University, Chandigarh, India

## Background

Lung innate immune response plays an important role in the clearance of pathogens (that is, bacteria, virus or fungi) from lungs. However, profound activation of innate immune cells (alveolar macrophages or neutrophils) can lead to development of acute lung inflammation or injury observed during pneumonia, acute respiratory distress syndrome (ARDS), or sepsis by producing various proinflammatory cytokines (that is, IL-1α, TNFα) and molecules (that is, hydrogen peroxide (H_2_O_2_)). Thalidomide is a drug, which has its own place in the history of medicine and has been used in various types of cancers and other chronic inflammatory disorders (erythema nodosum leprosum) but its mode of action as an immunomodulatory drug in acute infections (that is, pneumonia and sepsis) is not clear. Thus, the present study was designed to investigate its effect on pulmonary innate immune response during acute lung infection in BALB/c mice.

## Methods

Animals were divided into four groups (*n *= 20 for each group). Acute lung inflammation was induced by intranasal instillation of *Klebsiella pneumoniae *B5055 into mice without any anesthesia and treated with thalidomide (30 mg/kg/day/p.o.) or normal saline orally using a treatment schedule shown to modulate proinflammatory innate immune response. Various proinflammatory (IL-1α, TNFα) as well as anti-inflammatory (that is, IL-10) cytokines were estimated by ELISA. Pulmonary macrophage-mediated phagocytosis and bactericidal assays were performed. Alveolar macrophages were evaluated in terms of macrophage spreading ability assay. H_2_O_2 _production by macrophages was assessed according to the method based on the process of horseradish peroxidase-dependent oxidation of phenol red assay. Neutrophil infiltration to the lungs was determined by histopathological analysis.

## Results

Thalidomide treatment modulated proinflammatory function of alveolar macrophages by significantly decreasing their phagocytic potential in terms of phagocytic uptake and intracellular killing, spreading and H_2_O_2 _release. Besides that, thalidomide treatment also significantly decreased neutrophil infiltration into the lung alveoli. Remarkably, the levels of proinflammatory cytokines (IL-1α and TNFα) were found to be decreased significantly in the thalidomide-treated group but the levels of IL-10 were found to be significantly elevated.

## Conclusion

Thalidomide proved a promising immunomodulatory agent with a potential to modulate aggravated innate immune response observed during acute lung inflammation associated with pneumonia or sepsis caused by Gram-negative bacterial infection.

